# Cybervictimization among secondary students: social networking time, personality traits and parental education

**DOI:** 10.1186/s12889-019-7876-9

**Published:** 2019-11-11

**Authors:** Mónica Rodríguez-Enríquez, Miquel Bennasar-Veny, Alfonso Leiva, Maite Garaigordobil, Aina M. Yañez

**Affiliations:** 1 Developmental Psychology Department, University of Vigo, Ourense, Spain; 2 Nursing and Physiotherapy Department, University of the Balearic Islands, Palma, Balearic Islands Spain; 3 Research Group on Global Health & Human Development, Balearic Islands University, Mallorca, Spain; 4 Primary Care Research Unit of Mallorca, Balearic Islands Health Service, Mallorca, Spain; 5 Department of Personality, Assessment, and Psychological Treatments, Faculty of Psychology, University of the Basque Country, Leioa, Spain

**Keywords:** Cyberbullying, Cybervictimization, Personality, Adolescents, School, Social networking time

## Abstract

**Background:**

Cyberbullying among children and adolescents is a major public health concern. However, research has not yet definitively identified the risk factors associated with cybervictimization. The purpose of this study was to determine the association of cybervictimization with use of social networks, personality traits and parental education in secondary students.

**Methods:**

The study population consisted of 765 secondary students (56.5% girls) from Majorca (Spain) who were aged 15.99 years (grade 4). The data were from the 16 secondary school centers that participated in the ITACA Project, a multi-center, cluster randomized controlled trial. Cybervictimization was measured by the Garaigordobil Cybervictimization Scale, and the Big Five Questionnaire for Children was used to assess personality traits.

**Results:**

Results showed that 39.9% of the students were cybervictims. Univariate analysis indicated that more girls than boys were cybervictimized (43.1% vs 35.7%). Cybervictims spent more time in social networking sites than non-victims (6 h 30 min vs. 5 h 16 min) and had greater emotional instability (0.16 vs. -0.23) and extraversion (0.11 vs. -0.09) and were less conscientious (− 0.001 vs. 0.20). Multivariable analysis indicated that social networking time was not significantly associated with cybervictimization after controlling for personality traits, but the same personality traits remained significantly associated.

**Conclusions:**

Our findings indicate that cyberbullying is a frequent and relevant problem in adolescents. Big Five personality traits are related with cybervictimization. Possible ways to design interventions include promoting social leisure activities, encourage responsible attitudes and provide stress coping tools.

## Background

Bullying via online media, especially social networking sites (SNSs), has emerged as a major problem in recent years [1]. SNSs and other new information and communication technologies provide a wide range of opportunities for communication with others, but there are also certain risks. Not surprisingly, most people (including bullies) use SNSs in a similar manner and with similar purposes as face-to-face communication. Bullying among schoolchildren that occurs through online technologies is considered “cyberbullying” and the victims are considered “cybervictims” [2]. The characteristics of cyberbullying are similar to those of traditional bullying, in that both are acts of intentional aggression by one or more individuals that repeatedly target people who cannot easily defend themselves. However, because cyberbullying occurs via electronic media, it can also provide anonymity, disinhibition, and a larger audience [3]. These unique characteristics of cyberbullying may explain the stronger association of cyberbullying than traditional bullying with suicide [4].

The estimated prevalence of cybervictimization is approximately 10 to 40% [5–7]. However, it can be difficult to compare the prevalences of cybervictimization in different populations because there is no consensus on the specific parameters that define cyberbullying and cybervictimization, and because there are very few reliable screening instruments. Thus, it is likely that differences in the study populations, time frames, and methodologies of previous studies explain their different results [8].

The general aggression model propose personality as an important factor to understanding personal factors that influence aggressive behavior [9]. Personality, personal factors (gender, age, personality, socioeconomic status, technology use, values and perceptions) and situational factors (perceived support, parental involvement and school climate) may predispose a young person to become a cybervictim. Moreover, some these factors may be interrelated [8].

Adolescents aged between 12 and 15 years have the highest risk for cybervictimization, and this risk gradually declines beginning at age 16 [10, 11].

Research on gender differences in cybervictimization showed controversial results and the majority of studies did not find any differences [12]. However, some studies found a higher prevalence of cybervictimization among girls than boys [7, 13–15]. It has been previously reported that boys tend to get involved in direct forms of physical or verbal aggression [16] and girls use indirect aggression, like spreading rumors or social exclusion [17]. However, a more recent transcultural study with adolescents from six countries, including Spain, found more boys using indirect methods of aggressions than girls [18].

There is no agreement on the effect of socioeconomic status (SES) on cyberbullying. A large study of cyberbullying in Sweden found that children with at least one parent who had a college education were less likely to be victims than children whose parents did not have college education [19]. Likewise, some research indicates that public school students with low SES were more likely to report cybervictimization than private school students with high SES [20]. However, other research suggests that income level and the public/private nature of the school had no significant impact on cybervictimization [21].

Traditional bullying has affected all generations but cyberbullying mainly affects ‘digital natives’ (those born after 1980). Digital natives use the new technologies and SNSs as integrated tools in their everyday lives, because they provide opportunities for education, social interactions, and self-identity. Spending a greater amount of time on SNSs has been consistently associated with cyberbullying. Young Romanians, Poles and Germans who spend more time on social networks are at greater risk of being cybervictimized than those who spend less time [5]. The use of online forums and blogs has been associated with cybervictimization among Turkish adolescents [20, 22]. Time spent online is also positively associated with cybervictimization among US adolescent internet users [23, 24] and college students [25]. Moreover, young people who use mobile devices more frequently are more likely to be involved in cyberbullying than other students [26]. In this way, preventive counter-cyberbullying efforts have been aimed at reducing the time young people spend online [27]. However, longitudinal study in Spain found that cybervictimization predicted future problematic internet use (at 6 months), but problematic internet use did not predict future cybervictimization (at 6 months) [28]. Thus, the association between the use of new online technologies and cybervictimization appears to be complex, and there could be variables that confound these associations.

Similar to traditional bullying [29], cybervictimization is positively associated with emotional instability (feeling anxious and depressed, having low self-confidence, and use of maladaptive and/or impulsive strategies to cope with stress) [30] and introversion [31]. However, in contrast to traditional bullying, higher levels of extraversion may also be a risk factor for cybervictimization. Similarly, some studies have found an association of a low level of conscientiousness with being a victim of cyberbullying but not traditional bullying. Few studies have evaluated the relationship of the “Big Five Personality Traits” -extraversion, agreeableness, conscientiousness, openness, and emotional instability (neuroticism) - with cybervictimization.

Some individuals use SNSs and the internet as new ways to harass others, but it has not yet been established whether use of these resources themselves is a risk factor for cybervictimization. Based on the above research, we hypothesize that SNSs time will be positively related with cybervictimization. Moreover, personality traits and parental education could be confounders of the association between SNSs time and cybervictimization. Thus, the main purpose of the current study was to analyze if there is an independent relationship between cybervictimization and SNSs use, personality traits and parental education.

## Methods

This cross-sectional study analyzed the association of cybervictimization with use of SNSs and personality traits of Spanish secondary students.

### Participants and procedures

Participants were students who participated in the ITACA project (aged 15–16 years), a prospective multi-center, cluster-randomized controlled trial that aims to reduce the prevalence of smoking among secondary students. The initial ITACA sample consisted of 1708 students aged 11–12 years (in 2010 and 2011) from 16 Spanish secondary schools [32, 33]. Each school was randomly assigned to a 4-year curriculum-based multifactorial intervention group or a control group. We assumed a prevalence of cybervictimization of 15% among students 15–16 years old students [6], to obtain an 80% power to detect at least 45% differences in cybervictimization between categories of parental education, SNSs use or personality trait. We invited 17 schools to participate and 1080 students were surveyed (May to June of 2015), of those 765 completed the questionnaire.

The surveys were administered by two trained data collectors during a 45-min class. Students completed surveys in grade 4 of their secondary education. To ensure that the responses were confidential the teachers were asked to leave the classroom during the surveys.

### Measures

#### Cybervictimization assessment

Garaigordobil Cybervictimization Scale, an instrument validated in the Spanish population with a high validity and reliability [30], was used to evaluate cybervictimization. This scale also showed a high internal consistency (α = 0.83) in the present study. The cyberbullying behaviors were assessed by asking students about the frequency that they suffered 15 cyberbullying behaviors (spreading photos or videos of embarrassing situations, sending offensive and insulting messages, recording an assault and uploading it to the internet, making offensive phone calls, taking stolen photos and spreading them online, etc.). Neither a cyberbullying definition nor the word “bullying” or “cyberbullying” were given. The students who suffered one or more of these harassment behaviors, at less “sometimes” during the previous year, were identified as cybervictims.

#### Personality traits

The Big Five Questionnaire for Children (BFQ-C) [33], a 65-item questionnaire based on the five factors model was used to asses personality. Each of the five dimensions is evaluated by 13 items. The five basic personality traits were: openness, conscientiousness, extraversion, agreeableness and emotional instability (neuroticism). Openness, which refers to imagination or intellectual curiosity, especially in the school domain, and broadness or narrowness of cultural interests and fantasy/creativity. Conscientiousness deals with attention, willingness to work hard and fulfilling of commitments. Extraversion evaluates characteristics such as enthusiasm, assertiveness, activity or facility with other people. Agreeableness concerns empathy or kindness. Emotional instability assesses feelings of anxiety, depression, discontent, and anger. The Spanish adaptation of this questionnaire [34] includes all 65 questions, and each question was scored using a Likert-type scale that ranged from 1 (almost never) to 5 (almost always). Scores on each of five personality dimensions were computed by summing the responses to the items. The reliability of each personality traits was assessed by Cronbach’s Alpha, and these values were satisfactory for conscientiousness (0.87), extraversion (0.77), openness (0.82), instability (0.77), and agreeableness (0.71). Previous studies showed good psychometric properties of the BFQ-C [35].

#### Use of SNSs and screen time

Young people were asked to report the number of hours per day (weekdays and weekends) in which they used social networks, and their total screen time. These data were recorded as two standardized variables: daily screen hours and daily SNS hours.

#### Parental education

For each student, the education level of both parents was recorded. The four categories were: a) less than primary education (< 6 years); b) primary education (6–8 years); c) secondary education; and d) university studies. For analysis of these data, a dichotomous variable was used, in which “0” indicated groups (a) and (b), and “1” indicated groups (c) and (d).

### Statistical analysis

The prevalence of cybervictimization and descriptive data (sociodemographic variables, personality traits, use of SNSs, and screen time) were determined for the whole sample, and separately for cybervictims and non-victims. In the bivariate analysis, a chi-square test was used to assess the association of sex and parental education with cybervictimization, and a *t*-test was used to assess the association of student age, personality traits, use of SNSs, and screen time with cybervictimization. Then, a multivariate analysis (multivariate logistic regression) was used to further examine these relationships, with adjustment for possible confounding. All analyses were performed on SPSS 22.0.

## Results

A high percentage (39.9%) of all adolescents reported being victims of some type of cyberbullying behavior during the last year (Table 1). A comparison of cybervictims with non-victims indicated no significant difference in age (overall mean: 15.99 ± 0.05 years), but that girls were more likely to be cyberbullied than boys (43.1% vs. 35.7%, *p* < 0.05). The parents of students in the two groups had no significant differences in education, and about half of the parents overall had secondary education or higher. No significant associations between cybervictimization and parents’ educational level (mother’s *p* = 0.10; father’s *p* = 0.72) and age (cybervictims 14.95 ± 0.08 vs non-cybervictims 15.03 ± 0.06; *p =* 0.11) were found. The cybervictims had significantly greater daily screen time (6 h 15 min ± 11 min vs. 5 h 16 min ± 10 min; *p* = 0.008) and daily time on SNSs (6 h 30 min ± 26 min vs. 5 h 16 min ± 20 min; *p* = 0.002). In addition, girls spent significantly more time in SNSs than boys (6 h 5 min ± 16 min vs. 5 h 3 min ± 20 min; *p* = 0.02). The cybervictims had higher scores for extraversion (0.11 ± 1.03 vs. -0.09 ± 0.95; *p* < 0.01) and emotional instability (0.16 ± 1.02 vs. 0.23 ± 0.90; *p* < 0.001), and lower scores for conscientiousness (0.001 ± 1.004 vs. 0.2 ± 0.96; *p* < 0.01).

**Table 1:** Characteristics of students who were and were not victims of cyberbullying

	Total Sample *n* (%) / Mean (SD) *n* = 765	Victims *n* (%) / Mean (SD) *n* = 305	Non-Victims *n* (%) / Mean (SD) *n* = 460	*p*-value ^a)^	Effect Size^b)^
Age	14.99 (0.66)	14.95 (0.67)	15.03 (0.65)	0.110	
Sex				**0.040**	−0.074
Female	432 (56.5%)	186 (43.1%)	246 (56.9%)		
Male	333 (43.5%)	119 (35.7%)	214 (64.3%)		
Mother’s education				0.096	
Less than primary	23 (4.4%)	12 (5.9%)	11 (3.4%)		
Only Primary	111 (21.1%)	52 (25.5%)	59 (18.4%)		
Secondary	225 (42.9%)	79 (38.7%)	146 (45.5%)		
University	166 (31.6%)	61 (29.9%)	105 (32.7%)		
Father’s education					
Less than primary	26 (5.1%)	11 (5.5%)	15 (4.8%)	0.717	
Only Primary	122 (23.8%)	46 (23.0%)	76 (24.4%)		
Secondary	261 (51.0%)	107 (53.5%)	154 (49.4%)		
University	103 (20.1%)	36 (18.0%)	67 (21.5%)		
Social network use and screen time					
Daily screen time, min	338 (288)	375 (306)	316 (274)	**0.008**	0.203
Daily screen minutes, z-score	0 (1)	0.12 (1.06)	−0.076 (0.95)		
Daily social networks, min/week	338 (359)	390 (371)	308 (349)	**0.002**	0.228
Daily social networks minutes, z-score	0 (1)	0.14 (1.035)	−0.86 (0.97)		
Personality traits, z-score					
Openness	0 (1)	0.023 (1.024)	−0.021 (0.990)	0.561	
Conscientiousness	0 (1)	−0.124 (1.019)	0.080 (0.976)	**0.007**	0.314
Extraversion	0 (1)	0.131 (1.041)	−0.074 (0.955)	**0.006**	−0.207
Agreeableness	0 (1)	0.019 (1.044)	−0.007 (0.960)	0.730	
Emotional Instability	0 (1)	0.240 (1.045)	−0.167 (0.928)	**< 0.001**	−0.417

^a)^ Student’s t-test or Chi-square test. ^b)^ Effect size were estimated as Cramer’s V for categorical variables or d-Cohen’s d for continuous variables

A multivariate analysis indicated the presence of collinearity between screen time and SNSs time, so we eliminated screen time, but included the other variables in the three multivariable analyses (Table 2). The first model analyzes the association of cybervictimization with sociodemographic and personality variables (excluding SNS time). The results show that age (OR = 1.31, 95% confidence interval: 1.01–1.69), conscientiousness (OR = 0.73, 95% confidence interval: 0.60–0.89), extraversion (OR = 1.45, 95% confidence interval: 1.19–1.78), and emotional instability (OR = 1.58, 95% confidence interval: 1.32–1.89) were significantly associated with cybervictimization. The second model analyzes the association of cybervictimization with time on SNSs (excluding personality traits). The results show a significant association between time on SNSs and cybervictimization (OR = 1.21, 95% confidence interval: 1.04–1.41). The third model considered all the variables of the two previous models. The results show that time on SNSs was not significantly associated with cybervictimization (OR = 1.11, 95% confidence interval: 0.94–1.31). However, cybervictimization was significantly associated with conscientiousness (OR = 0.74, 95% confidence interval: 0.61–0.91), extraversion (OR = 1.42, 95% confidence interval: 1.15–1.74), and emotional instability (OR = 1.57, 95% confidence interval: 1.31–1.88).

**Table 2:** Multivariable analysis of the association of student characteristics with cybervictimization

	Model 1^a)^ aOR (95% CI)	Model 2^b)^ aOR (95% CI)	Model 3^c)^ aOR (95% CI)
Age	**1.305** (1.010–1.687)	1.197 (0.947–1.513)	1.276 (.985–1.653)
Female	1.268 (0.882–1.822)	1.331 (0.980–1.809)	1.231 (0.854–1.774)
Parental education			
Mother education (secondary or more)	1.060 (0.695–1.617)	1.010 (.693–1.474)	1.080 (0.707–1.651)
Father education (secondary or more)	1.023 (0.693–1.509)	1.098 (0.772–1.562)	0.990 (0.669–1.465)
Personality traits, z-score			
Openness	1.070 (0.901–1.271)	_	1.074 (0.903–1.279)
Conscientiousness	**0.730** (0.599–0.888)	_	**0.742** (0.606–0.908)
Extraversion	**1.454** (1.190–1.778)	_	**1.418** (1.154–1.742)
Agreeableness	1.035 (0.835–1.284)	_	1.037 (0.834–1.289)
Emotional Instability	**1.575** (1.318–1.881)	_	**1.566** (1.307–1.877)
Social network use			
Daily social network use		**1.211** (1.042–1.407)	1.109 (.938–1.311)

Logistic regression analysis; ^a)^ Model 1: X^2^ = 57.70; p > 0.001; −2loglikelihood = 845.51; Nagelkerke R^2^ = 0.08 ^b)^ Model 2: X^2^ = 14.40; *p* = 0.006; −2loglikelihood = 978.58; Nagelkerke R^2^ = 0.03; ^c)^ Model 3: X^2^ = 60.53; *p* > 0.001; −2loglikelihood = 849.41; Nagelkerke R^2^ = 0.12

## Discussion

The present study examined the impact of personality traits and sociodemographic characteristics on the association between cybervictimization and use of SNSs. Nearly 40% of the students in our study population reported cybervictimization during the last year. This result is similar to previous studies [5, 23], but somewhat higher than a similar study in Spain [6]. Thus, a major result of our study is that adolescents aged 15–16 years have a significant risk of being cybervictims.

Similar to other studies, we found that more girls than boys were cybervictims [36]. It is possible that girls use cyberbullying more than traditional bullying because it is a form of relational aggression (which is more common among girls) or because girls spend more time in SNSs. We favor the second possibility because our data indicate that girls spent significantly more time on SNSs than boys.

Our results indicated that parental education was not significantly associated with cybervictimization, in contrast to the findings of Låftman et al. in Sweden [19]. Thus, the level of parental education in Spain appears to have a small impact on cybervictimization of adolescents in Spain than Sweden. It would be interesting to examine the impact of parental skills regarding online safety on protection of their children from cybervictimization.

Unlike other studies [28, 37–39], our results do not support the presence of an independent association between SNSs time and cybervictimization. Instead, our results indicate that adolescents who have high scores for neuroticism or extraversion, or a low score for conscientiousness have a greater risk of cybervictimization. Our multivariable analysis indicates that youths who spend more time in SNSs, but do not have these personality traits, had no greater risk of being cybervictims. One explanation is that students who scored high on extraversion and neuroticism, but low on conscientiousness, may engage in more risky online behaviors (such as uploading photos or comments about drugs, alcohol, sex, parties, and nudity). Students who score high on neuroticism are more likely to feel negative emotions and use maladaptive methods to cope with stress and may therefore express their discomfort more openly on SNSs. Cyberbullies could easily exploit these disclosures and harass these individuals.

Extroverted people are energized, talkative, and enthusiastic, and they enjoy social activities and parties. They are also more prone to openly share their thoughts and emotions and take pleasure in public demonstrations. However, sharing of personal information in SNSs can be a problem, because it becomes available for exploitation by cyberbullies [40].

A high score on conscientiousness indicates a serious, formal, cautious, motivated, and organized person, who usually has high academic achievements. Other students may feel envious of the successes of these conscientious youths, and target them by cyberbullying [41, 42]. However, we found and inverse association between conscientiousness and cybervictimization; in other words, adolescents with low scores for conscientiousness were more often the targets of cyberbullying. This may be because cautious and serious students are more careful about what information they share on SNSs. Thus, high conscientiousness could increase the risk for being a victim of traditional bullying but decrease the risk for being a victim of cyberbullying.

Our results suggest that the time on SNSs does not impact independently cybervictimization, although the way that online time is used may have an impact. In particular, our findings showed that high scores on neuroticism and extraversion, and a low score for conscientiousness could be associated with risky behaviors on social networks, and this, in turn, could lead to cybervictimization. To our knowledge, this is the first study to investigate the association of cybervictimization with use of SNSs that controlled for BFQ-C score. However, our study has some limitations. First, this is a cross-sectional study, so we cannot make conclusions regarding causality. In particular, although personality traits are relatively stable throughout an individual’s lifetime, including adolescents, we cannot confirm cause-and-effect relationships [43]. Furthermore, we identified a cybervictim as any individual who suffered harassment “sometimes” during the previous year. This cut-off point may be overly sensitive, because it identified individuals who only suffered from infrequent harassment as cybervictims. Moreover, neither a cyberbullying definition nor the word “cyberbullying” were given to avoid underreporting and labeling. Finally, this study focused on SNSs, although previous studies examined overall internet use. Although this could be a limitation of our study, we believe that effect of internet use on cyberbullying should be reflected by use of SNSs, because they facilitate social interactions.

## Conclusions

Our findings have important implications. School and parents should take in account the role of personality on adolescent’s risks behaviours. There is an effect of neuroticism and extraversion on cybervictimization. Furthermore, our findings contribute to understand how personality traits impact on cybervictimization and time of SNS suggesting possible ways to design personality traits-centered intervention. For example, promoting healthy social leisure activities from schools to meet the needs of more extroverted students, encourage responsible attitudes and provide stress coping tools could be strategies that could contribute to reduce both time of SNSs and cybervitimization in our environment. However, further research is needed to investigate if personality traits-centered interventions could prevent cybervictimization.

In conclusion, cyberbullying is common among secondary students in Spain, and certain personality traits were significantly associated with cybervictimization. The association between cybervictimization and time spent using SNSs may be explained as due to a confounding of certain personality traits with time of SNS use.

## Data Availability

The datasets used and/or analysed during the current study are available from the corresponding author on reasonable request.

## Abbreviations

BFQ-C: Big Five Questionnaire for Children
SES: Socioeconomic Status
SNSs: Social Networking Sites
